# Pediatric Sickle Cell Disease and Stroke: A Literature Review

**DOI:** 10.7759/cureus.34003

**Published:** 2023-01-20

**Authors:** Taral Parikh, Ashish Goti, Kanica Yashi, Naveen Prasad Gopalakrishnan Ravikumar, Narendrasinh Parmar, Nilesh Dankhara, Vimal Satodiya

**Affiliations:** 1 Pediatrics, Hamilton Health Center, Harrisburg, USA; 2 Pediatrics, Tulane School of Medicine, New Orleans, USA; 3 Internal Medicine, Bassett Health Care, Cooperstown, USA; 4 Internal Medicine/Nephrology, Oregon Health & Science University School of Medicine, Portland, USA; 5 Pediatrics, East Tennessee Children's Hospital, Knoxville, USA; 6 Pediatrics and Neonatology, University of Mississippi Medical Center, Jackson, USA; 7 Pediatrics, Omni Family Health, Bakersfield, USA

**Keywords:** transcranial doppler, hydroxyurea, pediatric sickle cell disease, headache, cerebrovascular disorders

## Abstract

Both ischemic and hemorrhagic strokes in children can be a complication of sickle cell disease, which also affects adults. The occurrence is high without any screening or preventative care. This review article found that although transcranial Doppler (TCD) has reduced the prevalence of stroke in pediatric patients, there is still a need for an epidemiological survey to define such screening for adults, the ideal dose of hydroxyurea to reduce the incidence of stroke, and to identify silent cerebral stroke to prevent its complications. Increased hydroxyurea prescription and specific antibiotic and vaccination regimes lowered the occurrence of this condition. In pediatric cases with a time-averaged mean of the maximal velocity greater than 200cm/s, transcranial Doppler screening and preventive chronic transfusion for at least the first year have lowered the occurrence of stroke by up to 10 times. The ideal dose of hydroxyurea is still debatable, but it seems to reduce the risk of the first stroke to a comparable level in the average population. Adult ischemic and hemorrhagic stroke prevention has not yet received the same attention. Though there are fewer studies, sickle cell disease is also more common than age-matched controls in terms of silent cerebral infarction on magnetic resonance imaging (MRI), as well as other neurological problems such as cognitive impairment, seizures, and headaches. Currently, there is no evidence-supported way to prevent ischemic stroke in adults at any age. Also, there is no defined ideal dose of hydroxyurea that can be helpful in preventing strokes. Data also lack a way to identify a silent cerebral infarction, so its complications can be prevented. An additional epidemiological survey may help in the prevention of the condition. The primary aim of this article was to emphasize the importance of information on clinical, neuropsychological, and quantitative MRI assessment of sickle cell patients to understand the epidemiology and etiology of stroke in sickle cell patients to prevent stroke and its related morbidity.

## Introduction and background

Sickle cell disease (SCD) is now prevalent worldwide due to the migration of people of various types of genetic makeup across the globe. This has been observed in areas where malarial outbreaks are common [1]. Stroke is a recognized complication of homozygous sickle cell anemia (SCA; hemoglobin SS-HbSS) [2] and may occur in compound heterozygotes such as individuals who have HbSC illness and HbS-thalassaemia (HbS-that) [2,3]. The lack of clarity on the diagnostic inclusion criteria is a significant challenge. Acute MRI techniques frequently discriminate between a wide range of generalized and focal non-vascular and vascular diseases, which have important therapeutic implications. In addition to stroke, critical neurological signs and symptoms that are common in SCD include transient ischemic attack (TIA), seizures, headaches, and coma [4-7]. Several situations, such as infections, acute chest syndrome (ACS), acute anemia, after surgery, transfusions, immunosuppression, or seemingly spontaneously, can result in altered mental status with or without an abridged level of consciousness, headache, seizures, visual loss, or focal signs. For instance, 3% of patients with SCD and ACS presented with neurological symptoms while another 7-10% of patients with ACS have acquired neurological symptoms [8,9]. Although this may not be the case when coding using the International Classification of Diseases, these patients were classified clinically as having experienced a cerebrovascular accident (CVA) [10].

## Review

Stroke definition and general information

A focused neurological impairment lasting more than 24 hours is what the World Health Organization defines as a clinical stroke. Clinically, TIAs are defined as focal neurological impairments that last less than one day, even though acute neuroimaging may show abnormalities. Both symptomatic and asymptomatic cerebral infarctions (silent or covert) are possible. Sickle cell disease (SCD) is often seen as the reason for stroke in children in areas where the syndrome is common such as Africa, Europe, and the USA. With age-related increases in prevalence and incidence, adults with SCD are also affected. Less than 10% of SCD subjects experience their first stroke during infancy or adolescence without screening and preventative care, with a quarter experiencing one in middle age. The lifetime risk of stroke is almost one-third with SCD [11-15].

Stroke mortality in SCD

Stroke is a significant source of mortality in SCD and was responsible for 10% of autopsy cases from 1929 to 1996. This number dropped to 4% during the Howard University period from 1976 to 2001. The Co-operative Study of Sickle Cell Disease (CSSCD) found no link between ischemic stroke and death. Still, a series of hospitalized Californian subjects with ischemic stroke saw 7% of patients pass away as compared to SCD (30%) and hemorrhagic stroke (26%) in this study. In the Californian trial, 5% of children with SCD hospitalized for a first, or subsequent, stroke passed away [16,17].

Prevalence of all strokes in people with SCD who do not get prophylaxis

Early research on strokes in children revealed that approximately 75% of cases were ischemic, and the other 25% were hemorrhagic. At the same time, more recent data point to a more significant percentage of ischemic strokes. Significantly, there seems to be considerable overlap in the risk variables for both hemorrhagic and ischemic stroke when using current neuroimaging for screening and diagnosis. In a USA study, the general incidence of pediatric stroke was 0.0129/100 patient-years of observation while ischemic and hemorrhagic stroke incidences were 0.0058 and 0.007, respectively. SCD was the most frequent cause of stroke (39%), with incidence rates of ischemic and hemorrhagic strokes of 0.285/100 person-years of observation (PYO), 0.238 (0.078-0.556), and 0.0475 (0.012-0.266) per 100 PYO, respectively. Stroke incidence has increased 221-fold in children with SCD, 41-fold in ischemic stroke cases, and seven-fold in hemorrhagic stroke cases [18]. Hemorrhagic strokes are more common in adults, and the prevalence of these strokes may be underreported because cerebral bleeding may result in rapid death in a hospital [19-20].

CVA prevalence in all types of SCD (HbSC, HbSS, and HbS-thalassemia) at the time of entry to the CSSCD was <4% overall, ranging from 11/1000 in children under two years old to 76/1000 in people in their 40s and 50s. In a 411-child study conducted in Brazil, the overall prevalence of overt stroke ranged from 3.1% to 7.2% overall [21]. The majority in France was 3.2% for HbSS, 1.2% for HbSC, and 3.8% for HbS-thalassemia [22]. The overall prevalence of stroke was 4.2% at the time of enrollment in the German registry and 5% in individuals with HbSS [23], compared to 4.5% (1/22) in one study [24] and 2.6% at the time of enrollment in a subsequent, more extensive Spanish registry [25]. In the East London Cohort Study, the prevalence of overt stroke was 3.8% at baseline in 1991 (6 of 160) [19]. The overall rate of stroke was 3.8%, and the rate of cerebrovascular symptoms was 8.1% [26].

In a subsequent study, she found an 8.4% prevalence using the WHO criteria for stroke and TIA with CT scans for all patients [27]. Intriguingly, stroke and paraplegia were more common in a comparative study in Nigeria than in healthy controls [28]. It is unclear whether acquired diseases of the white matter, spinal strokes, or premature birth caused paraplegias. According to a Gulf state study of the reported homozygous SCD prevalence rates, stroke seems infrequent in Arab states (1.8%) [29]. In Kuwait, the prevalence of stroke is 1.4% in children [30], and in Shiraz, Iran, it seems to be low among children with SCD. When adults are considered, the stroke prevalence is high in Saudi Arabia's provinces (~7%) [31-34].

Ischemic stroke

It is common for children between the ages of two and 10 years to experience an overt ischemic stroke. The typical presentation is hemiparesis, and without subsequent prophylaxis, recurrence occurs in up to 67% of cases. Among those aged 20-29 years, the incidence of ischemic stroke falls to its lowest point; however, it then peaks beyond age 35. Incidence rates vary significantly between developed and developing nations. The prevalence of stroke in children increases with delayed diagnosis. Therefore, neonatal screening and avoiding comorbidities such as acute anemia and infection may decrease the risk of stroke. In normal pediatric subjects, meningitis secondary to Haemophilus influenzae and Streptococcus pneumonia may be related to cerebrovascular illness and later stroke [35]; however, it is debatable whether this has been a significant cause of SCD. In some centers, newborn SCD screening began in the 1980s [36,37]. Around the same time, penicillin prophylaxis for young infants was introduced [38], and shortly after that, vaccination against Haemophilus influenzae and Streptococcus pneumonia was initiated. In various areas, stroke incidence in children appears to be reduced, and these advancements seem to be linked to a drop in mortality [35-40].

Doppler transcranial ultrasound

Primary stroke prevention has been accomplished using TCD for stroke risk assessment and proper care for those at high risk. Intracranial arteries are commonly affected in SCD, and TCD is utilized to quantify the time-averaged mean of the maximum velocities (TAMMV) in these arteries. Increased TCD velocities may be related to arterial diameter narrowing or increased CBF in the presence of anemia. Extracranial post-bulb carotid stenosis can be identified using the submandibular technique [39,40].

When distal middle cerebral artery (MCA)/internal carotid artery (ICA) velocities are between 190 and 140 cm/s, stenosis in SCD may be observed on conventional angiography; rates greater than 190 cm/s are associated with significant arterial stenosis. In three years, an irregular TAMMV ICA/MCA of 200 cm/s was linked to a 40% increased risk of stroke, whereas conventional TAMMV was linked to a 7% increased risk [40]. Before therapy, the stroke incidence in the 1991-1998 East London trial was 12.7/100 PYO for those with irregular TCD and 1.94/100 PYO among those with conditional TCD. In the Ibadan cohort, the incidence of stroke was 11.1/100 PYO in those not treated for abnormal TCD [41].

Frequency of conditional and abnormal transcranial Doppler

Most screening programs begin at two years of age, and stroke ancillary to other mechanisms, such as embolus through a patent foramen ovale, may be significant in those younger than the above. Even in childhood, ICA/MCA velocities are greater than in healthy children. Nonetheless, those in the conventional range are infrequent and irregular velocities have not yet been recognized. In the 1990s, 8% of children in the USA and 6% of children in the UK had aberrant TCD. According to subsequent analysis, 18 (3%) of the 542 East London infants who underwent TCD screening had high abnormal >220 cm/s TAMMV, according to a subsequent analysis [42]. Conferring to the Stroke Prevention Trial in Sickle Cell Anemia (STOP) criteria, preliminary research in Paris indicated that 9.6% of infants had abnormal TCD, and a further examination of their newborn cohort saw that 25% of kids had abnormal TCD. Atypical TCD was present in two of the 48 (4%) Spanish infants with SCD. Data from the DISPLACE (Dissemination and Implementation Looking at the Care Environment) consortium recently showed a low rate of 2.9% and a median age of 6.3 years for the first aberrant TCD, although there were implementation issues across sites. Therefore, the stage at first TCD was often only marginally less than this. In Jamaica, it was 6.7%, however, some of these children had already experienced a stroke. In Nagpur, central India, of the 178 individuals examined, 3% had aberrant TCD, and 4.5% had conditional TCD. An earlier study in Tanzania estimated that 7% of TCDs were abnormal, but subsequent research discovered lower rates than those in other East African countries such as Kenya. As per Kirkham and Lagunju, Arabs with SCD had a higher prevalence of normal TAMMV than Caucasians, Africans, and people who had previously received an exchange transfusion [18]. As per Lagunju et al., the risk of recurrence of stroke remains high in the absence of preventive treatment [43].

Children with SCD and abnormal TCD are more likely to experience strokes because of chronic blood transfusions and TCD screening

The STOP study established that TCD is a valuable instrument for identifying children with SCD who are at peril of stroke and that regular blood transfusion lowered the risk, even though there is a significant difference over time within persons. TCD screening has not yet been proven valuable in identifying adults with SCD at risk for stroke. Children with TCD MCA or ICA TAMMV of 200 cm/s are at a high risk of having their first arterial ischemic stroke (10-13% each year) if they do not receive continued steady blood transfusion therapy for a prolonged period. The stroke frequency in the STOP trial's transfusion arm was 0.9 per 100 person-years of observation (PYO) as opposed to 10.7 per 100 PYO in the traditional arm [44]. People with conditional TCD studies also have an advanced danger of stroke compared to those with velocities of less than 170 cm/s.

Since the STOP study was terminated early due to a significant benefit in favor of blood transfusion, screening and blood transfusion have been suggested as the standard of care in the UK and the USA [45]. Stopping transfusions reportedly increased the risk of stroke and irregular velocities, which didn't occur in the transfusion arm, as there appears to be a strong link between TCD evaluation and routine transfusion for people with abnormal TCD and a much-decreased incidence of stroke. Stem cell transplantation's impact on children with SCD and abnormal TCD's risk of stroke: It is on a positive note by showing TAMMV was lower.

Children with SCD and normal TCD stroke syndromes

The stroke frequency was 1.18/100 PYO for TCD patients in the East London cohort who were labeled as always usual. After chest trauma, a teen with posterior reversible encephalopathy syndrome suffered white matter damage.

Intracranial hemorrhage

Intraparenchymal, intraventricular, subarachnoid, and spontaneous subdural hemorrhages have all been linked to SCD patients. Although hemorrhage affects children frequently, it most frequently affects adolescents (20-30 years) [2]. If a patient has previously experienced an infarction, their risk of bleeding increases as they age [17]. Subarachnoid and intracerebral hemorrhage can be brought on by recent transfusions, corticosteroid use, bone marrow transplantation, and acute hypertension. Particularly in the vertebrobasilar circulation, rupturing aneurysms frequently occur near the bifurcations of prominent veins. Blood loss may also be connected to reversible posterior leukoencephalopathy and venous sinus thrombosis. Epidural hemorrhages have been reported in SCD patients with no significant head trauma, most likely because of hypervascular bone areas. It is currently unknown whether treatments for SCD in children have affected how frequently hemorrhagic stroke occurs. The data, however, show that it has not, as there were 0.09 hemorrhagic strokes per 100 PYO for East London network patients who underwent TCD screening [18].

Seizures

Patients with SCD may experience both single and recurrent seizures. Since neuroimaging is prohibitively expensive in low- and middle-income nations, where the majority of the cohort data comes from, it is tough to discern between febrile and acute symptomatic seizures in young children. Sever percent to 10% of those with SCD will have at least one episode [44,46].

Headache

Approximately 20% to 45% of people with sickle cell experience headaches at any age, especially in young children. The proportion of SCD patients who reported headaches remained consistent in adults while increasing in teenagers for controls chosen from the university, even though the difference was statistically significant at all ages. Neuroimaging should be performed as soon as possible because a subdural, intraparenchymal, subarachnoid, or intraventricular cerebral hemorrhage can also present as a severe headache. As per Hebbel, in patients who report acute headaches, pseudotumor cerebral and venous sinus thrombosis have also been recognized and should be ruled out [47].

CNS infections

CNS infections, such as meningitis, bacterial abscesses, and cerebral tuberculoma, can also strike children with SCD. Penicillin prophylaxis and immunizations have helped reduce CNS infections in the USA. Still, these anticipatory measures are rarely made obtainable in Africa, where malaria is a significant cause of comparable illnesses.

Coma

Even though other conditions, such as extensive middle cerebral artery infarction with edema and midline shift, posterior reversible encephalopathy syndrome, which may progress to border-zone infarction, and venous sinus thrombosis can also present, coma in SCD may be produced by intracranial hemorrhage. In addition to dysphasia and gait problems, other markers of covert (or silent) cerebral infarction include soft neurological signals and dysphasia [48].

Additional neurological issues

Clinical accounts of myopathy, myelopathy, and neuropathy can be found even if it might not have been able to get a neurological opinion or undertake an examination involving electromyography, nerve conduction, and muscle biopsy.

Cognitive imbalance

It is challenging to draw accurate conclusions about the frequency of cognitive impairment, as studies rarely offer formal diagnoses. Many studies also report similar mean mental capacity, contrasting with the percentage of patients whose test results fall into recognized clinical groups. However, given that knowledge of the risk factors for cognitive damage will probably be essential in expanding treatments, it is vital to use caution when interpreting the outcomes of tests developed outside of Africa. According to research, >50% of adults and adolescents reported having issues with their ability to pay attention, exercise executive function, process information rapidly, and comprehend what they were reading. Patients are more likely than matched controls to develop clinically severe cognitive impairment, even though there is much heterogeneity among SCD groups. The most recent criteria recommend neurodevelopmental screening, particularly in the first several years of life [18].

Silent cerebral infarctions

It can occur by the sixth month of birth. Prevalence reaches 25% by age 6, 39% by 18, and 53% by the time a person is in their early 20s, according to data from the USA and Europe [48]. No reports of a plateau in prevalence have been made, and 37% of SCD patients had multiple lesions. Prevalence estimates can vary based on voxel size, scanner magnet strength, and age. When compared to children who do not have SCD, children with non-sickle stroke had a more significant rate of silent infarcts on MRI, which likely, at least in part, reflects the population's chronic vascular impairment. Many SCI patients have normal TCD and MRA, and other potential causes of SCI include emboli connected to right-to-left shunting. However, despite the possibility that Moya Moya syndrome or stenosis, intracranial cerebrovascular diseases, are related to silent infarction [49], many SCI patients also have normal TCD and MRA. A higher likelihood of abnormal psychometric testing may exist in SCD patients with overt stroke or SCI. However, a correlation between cognitive function and SCI has been harder to find in MRI studies with increasing field strength [49-50]. In addition to a skilled radiologist's examination of the traditional MRI data, quantitative MRI may help resolve some of these problems and offer relevant information to clinical practice.

Magnetic resonance angiography findings for cerebrovascular diseases

Compared to conventional angiography, MRA can be up to 85% accurate in diagnosing SCD. Young children with cerebrovascular disease can be identified with MRA. One investigation found three out of 29 infants aged seven to 48 months to have MRA abnormalities. Several risk factors for the main neurological and cognitive effects of SCD are similar, but not all, despite relatively small sample sizes and geographically distributed research. The three most prevalent haplotypes among Africans and African Americans are Senegal, Benin, and Bantu. Furthermore, Saudi Arabia and India each have a unique haplotype.

Standard management of sickle cell disease

Transfusion is the main treatment during an acute episode, and most adults have received at least one blood transfusion. It increases oxygen-carrying capacity, improves blood flow in severe anemia, and is helpful in acute episodes like splenic sequestration, aplastic crisis, stroke, acute chest syndrome, multiple organ failure, priapism, etc. Hydroxyurea increases the level of fetal hemoglobin and is clinically associated with reduced morbidity and mortality. It can be started in children as early as nine months, and a study showed that a median dose of 25.6 mg/kg/day successfully reduced painful crises and organ damage and was well-tolerated. New therapeutic agents, such as L-glutamine, was also approved for the prevention of painful crisis and vaso-occlusive events in patients with SCD based on a study by Abboud [51].

## Conclusions

In Africa, the problem of SCD is still pertinent, as there is a lack of governing bodies to help people with screening. There are also very few steps taken for the prevention and prophylaxis of subjects at risk of SCD. The socio-economic conditions of these regions may be the main reason for this prevalence. However, in economic countries, SCD is known to have been lowered due to various preventive measures. Currently, there is no evidence-supported way to prevent ischemic stroke in adults at any age or a defined ideal dose that can be helpful to prevent stroke. Data also lack a way to identify a silent cerebral infarct, so its complications can be prevented. The additional epidemiological survey may help in the prevention of the condition.

## References

[1] Piel FB, Patil AP, Howes RE (2010). Global distribution of the sickle cell gene and geographical confirmation of the malaria hypothesis. Nat Commun.

[2] Ohene-Frempong K, Weiner SJ, Sleeper LA (1998). Cerebrovascular accidents in sickle cell disease: rates and risk factors. Blood.

[3] de Montalembert M, Beauvais P, Bachir D, Galacteros F, Girot R (1993). Cerebrovascular accidents in sickle cell disease. Risk factors and blood transfusion influence. French Study Group on Sickle Cell Disease. Eur J Pediatr.

[4] Kirkham FJ, Datta AK (2006). Hypoxic adaptation during development: relation to pattern of neurological presentation and cognitive disability. Dev Sci.

[5] Vichinsky EP, Neumayr LD, Gold JI (2010). Neuropsychological dysfunction and neuroimaging abnormalities in neurologically intact adults with sickle cell anemia. JAMA.

[6] Ali SB, Reid M, Fraser R, MooSang M, Ali A (2010). Seizures in the Jamaica cohort study of sickle cell disease. Br J Haematol.

[7] Vichinsky EP, Neumayr LD, Earles AN (2000). Causes and outcomes of the acute chest syndrome in sickle cell disease. National Acute Chest Syndrome Study Group. N Engl J Med.

[8] DeBaun MR, Armstrong FD, McKinstry RC, Ware RE, Vichinsky E, Kirkham FJ (2012). Silent cerebral infarcts: a review on a prevalent and progressive cause of neurologic injury in sickle cell anemia. Blood.

[9] Obama MT, Dongmo L, Nkemayim C, Mbede J, Hagbe P (1994). Stroke in children in Yaounde, Cameroon. Indian Pediatr.

[10] Macharia AW, Mochamah G, Uyoga S (2018). The clinical epidemiology of sickle cell anemia in Africa. Am J Hematol.

[11] Earley CJ, Kittner SJ, Feeser BR (1998). Stroke in children and sickle-cell disease. Baltimore-Washington Cooperative Young Stroke Study. Neurology.

[12] Strouse JJ, Jordan LC, Lanzkron S, Casella JF (2009). The excess burden of stroke in hospitalized adults with sickle cell disease. Am J Hematol.

[13] Strouse JJ, Lanzkron S, Urrutia V (2011). The epidemiology, evaluation and treatment of stroke in adults with sickle cell disease. Expert Rev Hematol.

[14] Powars D, Wilson B, Imbus C, Pegelow C, Allen J (1978). The natural history of stroke in sickle cell disease. Am J Med.

[15] Balkaran B, Char G, Morris JS, Serjeant BE, Serjeant GR (1992). Stroke in a cohort of patients with homozygous sickle cell disease. J Pediatr.

[16] Fatunde OJ, Adamson FG, Ogunseyinde O, Sodeinde O, Familusi JB (2005). Stroke in Nigerian children with sickle cell disease. Afr J Med Med Sci.

[17] Njamnshi AK, Mbong EN, Wonkam A (2006). The epidemiology of stroke in sickle cell patients in Yaounde, Cameroon. J Neurol Sci.

[18] Kirkham FJ, Lagunju IA (2021). Epidemiology of stroke in sickle cell disease. J Clin Med.

[19] Saidi H, Smart LR, Kamugisha E, Ambrose EE, Soka D, Peck RN, Makani J (2016). Complications of sickle cell anaemia in children in Northwestern Tanzania. Hematology.

[20] Green NS, Munube D, Bangirana P (2019). Burden of neurological and neurocognitive impairment in pediatric sickle cell anemia in Uganda (BRAIN SAFE): a cross-sectional study. BMC Pediatr.

[21] Belisário AR, Nogueira FL, Rodrigues RS (2015). Association of alpha-thalassemia, TNF-alpha (-308G>A) and VCAM-1 (c.1238G>C) gene polymorphisms with cerebrovascular disease in a newborn cohort of 411 children with sickle cell anemia. Blood Cells Mol Dis.

[22] de Montalembert M, Guilloud-Bataille M, Feingold J, Girot R (1993). Epidemiological and clinical study of sickle cell disease in France, French Guiana and Algeria. Eur J Haematol.

[23] Kunz JB, Lobitz S, Grosse R (2020). Sickle cell disease in Germany: results from a national registry. Pediatr Blood Cancer.

[24] Gómez-Chiari M (2003). Sickle cell anemia: experience in a center. Anales de Pediatría.

[25] Cela E, Bellón JM, de la Cruz M (2017). National registry of hemoglobinopathies in Spain (REPHem). Pediatr Blood Cancer.

[26] Piel FB, Jobanputra M, Gallagher M, Weber J, Laird SG, McGahan M (2021). Co-morbidities and mortality in patients with sickle cell disease in England: a 10-year cohort analysis using hospital episodes statistics (HES) data. Blood Cells Mol Dis.

[27] Lagunju IA, Brown BJ (2012). Adverse neurological outcomes in Nigerian children with sickle cell disease. Int J Hematol.

[28] Kehinde MO, Temiye EO, Danesi MA (2008). Neurological complications of sickle cell anemia in Nigerian Africans—a case-control study. J Natl Med Assoc.

[29] Wali Y, Kini V, Yassin MA (2020). Distribution of sickle cell disease and assessment of risk factors based on transcranial Doppler values in the Gulf region. Hematology.

[30] Adekile AD, Al-Sherida S, Marouf R, Mustafa N, Thomas D (2019). The sub-phenotypes of sickle cell disease in Kuwait. Hemoglobin.

[31] Alsultan A, Al-Suliman AM, Aleem A, AlGahtani FH, Alfadhel M (2018). Utilizing whole-exome sequencing to characterize the phenotypic variability of sickle cell disease. Genet Test Mol Biomarkers.

[32] Alsultan A, Alabdulaali MK, Griffin PJ (2014). Sickle cell disease in Saudi Arabia: the phenotype in adults with the Arab-Indian haplotype is not benign. Br J Haematol.

[33] Alsultan A, Aleem A, Ghabbour H (2012). Sickle cell disease subphenotypes in patients from Southwestern Province of Saudi Arabia. J Pediatr Hematol Oncol.

[34] Alzahrani F, Fallatah AM, Al-Haddad FM, Khayyat ST, AlMehmadi WM, AlQahtani BG, Alamri RS (2021). Risk factors and complications among pediatric patients with sickle cell anemia: a single tertiary center retrospective study. Cureus.

[35] Pryde K, Walker WT, Hollingsworth C (2013). Stroke in paediatric pneumococcal meningitis: a cross-sectional population-based study. Arch Dis Child.

[36] Quinn CT, Rogers ZR, McCavit TL, Buchanan GR (2010). Improved survival of children and adolescents with sickle cell disease. Blood.

[37] Telfer P, Coen P, Chakravorty S (2007). Clinical outcomes in children with sickle cell disease living in England: a neonatal cohort in East London. Haematologica.

[38] Gaston MH, Verter JI, Woods G (1986). Prophylaxis with oral penicillin in children with sickle cell anemia. A randomized trial. N Engl J Med.

[39] Adams R, McKie V, Nichols F (1992). The use of transcranial ultrasonography to predict stroke in sickle cell disease. N Engl J Med.

[40] Adams RJ, McKie VC, Carl EM (1997). Long-term stroke risk in children with sickle cell disease screened with transcranial Doppler. Ann Neurol.

[41] Lagunju I, Sodeinde O (2012). Prospective measurement of stroke incidence in childhood sickle cell disease. Arch Dis Childh.

[42] Telfer P, Dwan K, Simmons A (2016). Transcranial Doppler screening in a regional care network for sickle cell disease in the United Kingdom. J Pediatr Hematol Oncol.

[43] Lagunju IA, Brown BJ, Famosaya AA (2011). Childhood stroke in sickle cell disease in Nigeria. J Pediatric Neurol.

[44] Galadanci NA, Abdullahi SU, Ali Abubakar S (2020). Moderate fixed-dose hydroxyurea for primary prevention of strokes in Nigerian children with sickle cell disease: final results of the SPIN trial. Am J Hematol.

[45] DeBaun MR, Jordan LC, King AA (2020). American Society of Hematology 2020 guidelines for sickle cell disease: prevention, diagnosis, and treatment of cerebrovascular disease in children and adults. Blood Adv.

[46] Scothorn DJ, Price C, Schwartz D (2002). Risk of recurrent stroke in children with sickle cell disease receiving blood transfusion therapy for at least five years after initial stroke. J Pediatr.

[47] Hebbel RP (1994). Sickle Cell Disease: Basic Principles and Clinical Practice. https://experts.umn.edu/en/publications/sickle-cell-disease-basic-principles-and-clinical-practice.

[48] Kapustin D, Leung J, Odame I, Williams S, Shroff M, Kassner A (2019). Hydroxycarbamide treatment in children with Sickle Cell Anaemia is associated with more intact white matter integrity: a quantitative MRI study. Br J Haematol.

[49] Melek I, Akgul F, Duman T, Yalcin F, Gali E (2006). Neurological soft signs as the stroke risk in sickle cell disease. Tohoku J Exp Med.

[50] Ballas SK (1998). 7 Sickle cell disease: clinical management. Baillieres Clin Haematol.

[51] Abboud MR (2020). Standard management of sickle cell disease complications. Hematol Oncol Stem Cell Ther.

